# Scorpion Toxins and Ion Channels: Potential Applications in Cancer Therapy

**DOI:** 10.3390/toxins12050326

**Published:** 2020-05-15

**Authors:** Rosa Amalia Dueñas-Cuellar, Carlos José Correia Santana, Ana Carolina Martins Magalhães, Osmindo Rodrigues Pires, Wagner Fontes, Mariana S. Castro

**Affiliations:** 1 Laboratory of Toxinology, Department of Physiological Sciences, Institute of Biology, University of Brasília, Brasília DF 70910-900, Brazil; raduenasc@gmail.com (R.A.D.-C.); carlosjcsantana@gmail.com (C.J.C.S.); bioana.11@gmail.com (A.C.M.M.); osmindo@gmail.com (O.R.P.J.); 2 Brazilian Center for Protein Research, Department of Cell Biology, Institute of Biology, University of Brasília, Brasília DF 70910-900, Brazil; wagnerfontes2@gmail.com; 3 Research Group on Immunology and Infectious Diseases, Department of Pathology, Faculty of Health Sciences, University of Cauca, 5th Street # 4-70, Popayán 190001, Colombia

**Keywords:** apoptosis, cancer, cell death, ion channels, scorpion toxins

## Abstract

Apoptosis, a genetically directed process of cell death, has been studied for many years, and the biochemical mechanisms that surround it are well known and described. There are at least three pathways by which apoptosis occurs, and each pathway depends on extra or intracellular processes for activation. Apoptosis is a vital process, but disturbances in proliferation and cell death rates can lead to the development of diseases like cancer. Several compounds, isolated from scorpion venoms, exhibit inhibitory effects on different cancer cells. Indeed, some of these compounds can differentiate between healthy and cancer cells within the same tissue. During the carcinogenic process, morphological, biochemical, and biological changes occur that enable these compounds to modulate cancer but not healthy cells. This review highlights cancer cell features that enable modulation by scorpion neurotoxins. The properties of the isolated scorpion neurotoxins in cancer cells and the potential uses of these compounds as alternative treatments for cancer are discussed.

## 1. Apoptosis

**Figure 1:**
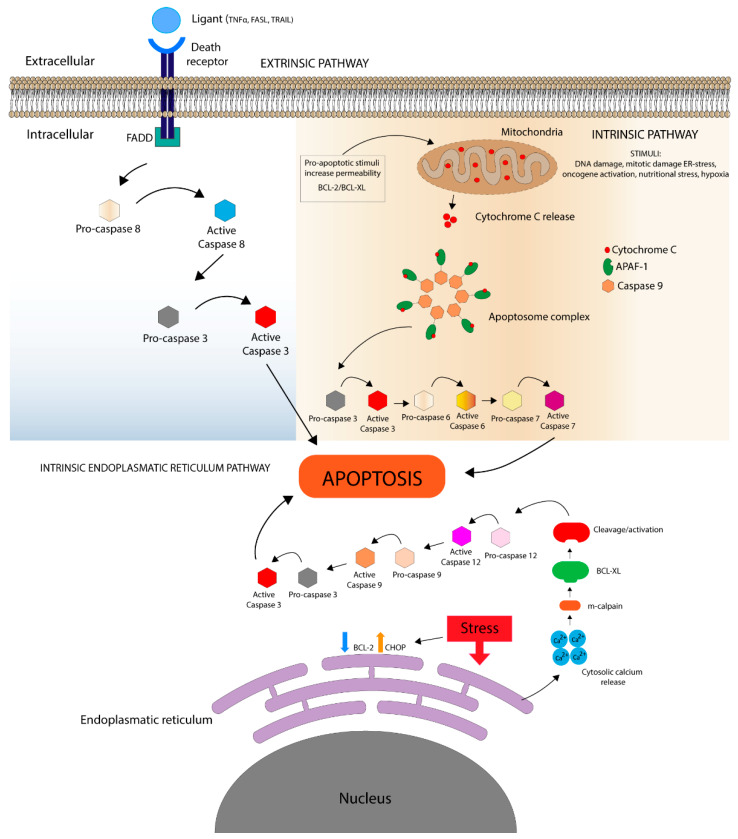
Main steps involved in apoptosis: extrinsic, intrinsic and intrinsic endoplasmic reticulum pathways.

Apoptosis, a genetically regulated normal physiological process of cell self-destruction, is marked, principally, by distinct morphological characteristics including cell shrinkage, cytoskeletal reorganization, pyknosis, DNA cleavage, and the formation of protrusions on the plasma membrane (blebs) [1]. Apoptosis can be triggered through three caspase-dependent signaling pathways. The death receptor and mitochondrial pathways are activated separately but converge at the end of the process [2,3] whereas the intrinsic endoplasmic reticulum pathway mechanism is not fully characterized, although it is distinguished by mitochondrial independence [4,5,6] (Figure 1).

There are also programmed cell death mechanisms, known as *apoptosis-like*, which are independent of caspases. These could be defense mechanisms to protect organisms against potentially harmful cells when caspase-mediated action fails. They may also be started in reaction to death stimuli or cytotoxic agents [7,8].

### 1.1. Death Receptor Pathways

Death receptor signaling is activated from the cell membrane when members of the tumor necrosis factor (TNF) superfamily and Fatty Acid Synthase (FAS) ligand family bind to their respective receptors. Ligand-receptor interactions start a cascade of reactions, including cleavage of procaspases into active caspases and triggering of apoptotic progression [2]. This apoptotic pathway principally involves activation of caspase-8, which leads to conversion of procaspase-3 into an active version [9]. Finally, tumor necrosis factor alpha (TNFα) can also induce apoptosis through an alternate pathway, namely by binding the receptor interacting protein (RIP), which leads to the activation of caspase-2 [10].

### 1.2. Mitochondrial Pathway

Also known as an intrinsic pathway, the mitochondrial pathway is dependent on death stimuli generated within the cell. Several initiators have been described including DNA damage, intracellular reactive oxygen species (ROS) increment, the unfolded protein response, and the lack of growth factors [2,11]. This pathway is regulated by mitochondria, which release intermembrane space proteins into the cytoplasm, due to increased permeability [12].

Among these proteins are cytochrome *c*, the second mitochondria derived activator of caspases (SMAC)/direct inhibitor of apoptosis (IAP)-binding protein with low pI (DIABLO), the apoptosis-inducing factor (AIF), endonuclease G (EndoG), and the high-temperature-requirement protein A2 (OMI/HTRA2) [2]. Cytochrome c binds to the apoptosis protease activating factor (Apaf-1) and forms a complex with dATP. Subsequently this complex promotes the conversion of procaspase-9 to caspase-9, mobilizing caspases 3, 6, and 7 [13,14].

#### Bcl Protein Family

The Bcl-protein (B-cell lymphoma 2) family is closely related to regulation of apoptosis via the mitochondrial pathway. Members of this family have proapoptotic and antiapoptotic capacities, and, by activating or inhibiting cell death, they maintain cell balances in different organs [15]. Bcl-2 is intimately related to the amount of cytochome *c*. Indeed, addition of proapoptotic Bcl-2 proteins to isolated mitochondria cause the release of cytochrome c, whereas over expression of Bcl-2 family members prevents it [16,17].

### 1.3. Intrinsic Endoplasmic Reticulum Pathway

The formation of protein aggregates through hydrophobic interactions can activate the endoplasmic reticulum (ER) stress response pathway. Aggregates can result from imbalances in calcium homeostasis, hypoxia or ischemia, oxidative stress, or uncontrolled increase of protein in the ER [6]. In these cases, the ER initiates cell death by activating caspase-12 [4]. This pathway operates independently of mitochondria and death receptors [8], in contrast to the two death pathways described previously.

## 2. Apoptosis in Cancer

Cancer is a public health concern around the world. GLOBOCAN 2018 estimated 18.1 million new cancer cases and 9.6 million cancer deaths in 2018 [18]. Furthermore, it is predicted there will be 23.3 million new cancer cases globally by 2030. Comparing to the number of cases in 2012, this represents 68% more cases, with moderately larger impact in low and medium human development index (HDI) countries (66% more cases in 2030 than 2012) than in high and very high HDI countries (56% more cases in 2030 than 2012) [19]. Many factors can promote the beginning of cancer, including physical, chemical, and biological factors that cause mutations and cause carcinogenesis. During carcinogenesis, apoptosis is one of the biological mechanisms that can be used to kill damaged cells and prevent neoplastic development. Therefore, defects in apoptotic processes that allow neoplastic cells or genetically unstable cells to survive can contribute to the pathogenesis of tumors [20].

Damage or changes to any component of apoptotic pathways can contribute to carcinogenesis. A prime example is regulation of the tumor suppressor p53. Normally, p53 promotes apoptosis, cell-cycle arrest, and DNA repair [21], but downregulation of p53 results in reduced apoptosis and enhanced tumor growth and development [22]. Another mechanism that inhibits apoptotic processes is related to a group of functionally and structurally similar Inhibitor of Apoptosis Proteins (IAPs), which regulate signal transduction, cytokinesis and apoptosis [23]. IAPs are endogenous inhibitors of caspases, which bind conserved *Baculovirus Inhibitor of apoptosis protein Repeat* (BIR) domains to the active sites of caspases. This interaction promotes active caspases degradation and prevents interaction with substrates [24]. Notably, caspase regulation plays important roles controlling apoptosis, and, in some cancers, the activity of caspases is diminished [25].

Most anticancer agents have not been designed for specific molecular or cellular targets, but they have been identified as apoptotic agents that inhibit proliferation of tumor cell lines [26,27]. Indeed, some existing therapies promote apoptosis in tumors, including treatment with cancer chemotherapeutic agents [28]; radiation [29]; cytotoxic lymphocytes [30]; hormone withdrawal or addition [31]; mild hyperthermia or ultra-low temperature [32,33]; and antibodies to the apo-1 or fas antigen [34] or HER2 antibody-drug conjugate [35]. Moreover, intervention in various gene regulatory pathways [36] and the use of nanopaticles for drug delivery in cancer cells [37] have been attempted.

## 3. Roles of Ion Channels in Apoptosis: Targets to Induce Cancer Cell Death

Chloride (Cl^−^), sodium (Na^+^), potassium (K^+^), and calcium (Ca^2+^) channels activation is involved in both cell proliferation and apoptosis. As ion channel inhibitors interfere with both cell proliferation and apoptosis, they appear to play active roles in the pathways that lead to replication and death [38]. Ion channels act in some stages of cancer and can mark progression via six main hallmarks, which cause (1) growth signals self-sufficiency; (2) cells not being affected by anti-growth signals; (3) resistance to apoptosis; (4) limitless replicative potential; (5) sustained angiogenesis; and (6) tissue invasion and metastasis [39]. These mechanisms facilitate the development of malignant cells and subsequent replication, thus contributing to tumor growth. Therefore, ion fluxes by ion channels are involved in apoptosis regulation [40], suggesting ion channels could be used as death regulatory tools to induce apoptosis and optimize anti-cancer treatments.

### 3.1. Voltage-Dependent Calcium Channels

Membrane depolarization is probably involved in limitless tumor cell proliferation, possibly by facilitating the entry of Ca^2+^ through voltage-dependent Ca^2+^ channels activation at higher voltages [41]. Among ion channels, Ca^2+^channels play critical roles in cell death mechanisms. Induced and physiological apoptosis that occurs through the mitochondrial-, cytoplasmic-, or ER-mediated pathways involve Ca^2+^ influx [42]. Moreover, Ca^2+^ entry into cells is necessary for cell cycle progression, and its reduction promotes the cell cycle to stop in the G1/S transition. When calcium channels are silenced, proliferation via the p53 tumor-suppressing transcription factor-dependent pathway is reduced, and upregulation of the cell-cycle arrest protein p21 is observed [39,43,44].

The expression of the Ca^2+^-selective TRPV6 channel was increased in primary tumors, and this has been associated with cancers of the epithelial origin such as of prostate, breast, pancreas, ovaries, endometrium, testicule, colon, and lung [45,46]. Dhennin-Duthille and collaborators showed that TRPV6 is overexpressed in invasive breast cancer cells and its selective silencing inhibited migration and invasion in the cell lines MDA-MB-231 and MCF-7 [47]. Due to the crucial role of TRPV6 in cancer cell proliferation, metastasis development and apoptosis inhibition, TRPV6 channel may be a novel target to be used as an effective therapy against cancers [46].

Activation of the cell death machinery in cancer cells by mitochondrial metabolism is closely related with the rates of Ca^2+^ [48]. Therefore, mitochondrial membrane permeabilization stimulation could be a promising therapeutic approach [49,50]. The mitochondrial permeability transition is caused by the opening of a large Ca^2+^ and oxidative stress-activated pore, the mitochondrial permeability transition pore: channel, which makes the inner mitochondrial membrane permeable to ions and solutes, leading to matrix swelling [51,52]. This mechanism is a usual cell death pathway enacted by some chemotherapeutics. The cell surface phosphatidylserine exposure is induced by a scramblase [53], which is activated by increased cytosolic Ca^2+^ [54,55].

### 3.2. Voltage-Dependent Potassium Channels

It is well established that K^+^ channels are critical determinants of cell membrane potential, and are thus important regulators of proliferation in different cells, including tumor cells [56]. The transmembrane potentials in cancer cells are more positive compared to healthy cells of the same histological origin [56,57]. Changes in the membrane potential of MCF7 cells during cell cycle progression have also been observed. The membrane potential of these cancer cells hyperpolarizes during the progression through G0/G1 into S phase. This change seems to be related to an increment in the permeability of the cell membrane to K^+^ [58].

K^+^ channels are also involved in apoptosis by regulating cell volume. When regulation fails and the outflow of K^+^ ions becomes too intense, water also leaves cells, diminishing cell volume and leading to the cell death [59]. There are over 70 types of K^+^ channels, but only a few are involved in external or internal stimuli that trigger apoptosis [60]. The promotion of apoptosis is associated with an increase in the K^+^ efflux [61], and it is an important mediator of early apoptotic cell shrinkage, caspase activation and DNA cleavage [62]. Caspase activation is considered the no return point in apoptosis and cellular K^+^ loss may be involved in this event [40,63].

K^+^ channels in tumor cells also function to block the growth and proliferation of cancer cells. There is evidence that Kv1.3, a voltage-gated K^+^ channel, is present in colon and breast cancer, while it is absent in normal human tissues [60,64]. Inhibition of ATP-sensitive potassium channels in human mammary carcinomas reversibly arrest cells in the G0/G1 phase of the cell cycle, inhibiting proliferation [65]. HERG-KCNE and Kv2.1-Kv9.3 channels blockers reduced proliferation of distinct uterine cancer cells [66]. More evidence comes from the expression of Eag1 (Ether-a-go-go 1, Kv10.1, or KCNH), a member of the potassium channel family. Eag1 expression is restricted to the brain in healthy adults, but it is also expressed in many tumor cells and tissues, including breast [67], lung [68], prostate [69], and human neuroblastoma [70]. The inhibition of Eag1 channels reduced cancer cell proliferation and also inhibited tumor growth *in vivo* [67]. This type of K^+^ channel could predict the transformation of cells and be used as a biomarker for early diagnosis of cancers [56]. Potassium channels blockers caused marked growth-inhibition of cancer cell lines, suggesting that potassium channels could serve as therapeutic targets [71].

### 3.3. Voltage-Dependent Chloride Channels

Cl^−^ channels regulate the flux of this anion and contribute to cellular membrane potential, transepithelial transport, and regulation of intracellular pH and cell volume [72]. There is evidence that high Cl^−^ conductance occurs during specific stages of the cell cycle [73]. Wondergem and colleagues showed that the blockage of the swelling activated by Cl^−^ current down-regulated hepatocyte proliferation [74]. Therefore, modulating Cl^−^ channel function could be important to cancer cells too.

Whole-cell patch-clamp measurements of tumor cells released from G0 indicate significant Cl^−^ channel activity during G1 and M phases of the cell cycle [75]. Kang and Kang [76] evaluated the effects of 1,3-Bis(2-chloroethyl)-1-nitrosourea (BCNU), the primary single-agent therapy used for treatment of human glioblastoma multiforme. Results showed that these cells overexpressed Cl^−^ intracellular channel 1 when compared with naïve cells, suggesting that chloride channels promote invasion and metastasis of gliomas and are involved in BCNU resistance [77].

Some voltage activated Cl^−^ channels are specifically expressed in cancer cells and not in normal cells [78]. For example, in glioma cell cultures, the chloride intracellular channel(CIC)-2 and ClC-3 are specifically upregulated [79]. Similarly, CIC-4 expression is increased in myofibroblasts that form stroma in breast cancers. CIC-4 expression is altered in several epithelial cancers, mainly in breast, kidney, and ovary [80].

The intra-tumor environment exhibits an acidic pH (as low as pH 5.6). The intense glycolytic activity in malignant cells is responsible for this acidification, affecting viability and proliferation of tumor cells [81]. Indeed, intracellular acidification promotes cellular damage and sensitizes cells to chemotherapy or hyperthermia. Apoptosis associated with intracellular acidification is controlled by Cl^−^ channels [82]. In some cases, acidic environments cause apoptosis by increasing caspase activity [83].

Cl^−^ channels blockers inhibit cell proliferation in different types of cells, including cancer cells [84,85]. Pharmacologic blockage of Cl^−^ channels interfered in cell proliferation by disrupting F-actin and causing osmotic swelling-induced cell cycle arrest [86,87]. Distilled water induced hypotonic shock in gastric cancer cell lines, MKN45 and Kato-III, inhibited volume decreases caused by Cl^−^ channel blockage [88]. Recently, it was demonstrated a decrease in lysosome stabilization induced by CLCN3 (chloride voltage-gated channel 3) suppression in glioma U251 cells treated with cisplatin [89].

### 3.4. Voltage-Dependent Sodium Channels

Na^+^ channels are expressed during the late phase of tumor progression, and can drive motility and regulate invasion and metastasis [85,90]. Na^+^ regulation is also closely related to cell volume, which is governed by the activity of the Na^+^, K^+^-ATPase. After an injury or sodium channel inhibition, cells become swollen because the amounts of Na^+^ increase sharply. Any condition that reduces or raises extracellular Na^+^, favors the accumulation of Ca^+2^, which, in turn, is related to cell death by necrosis [48]. Overloading can cause intracellular cytotoxicity and result in apoptosis, necrosis, or autophagy. Thus, cell death can be caused by loss of Ca^+2^ homeostasis [91].

Growing evidence has shown a relationship between the expression of voltage-gated sodium channels (VGSCs) in various cancers [92]. Nav1.4 and Nav1.7 channel isoforms are predominantly expressed in prostate cancer cell lines [93], whereas expression of Nav1.5 has also been recorded in lung cancer cells [94] and breast cancer cell lines [90,95]. This regulation may suggest the involvement of these Na^+^ channel isoforms in metastatic processes.

Studies using RT-PCR techniques identified three VGSCs, Nav1.5, Nav1.6, and Nav1.7, in two breast cancer cell lines, MDA-MB-231 and MCF-7. The overall level of VGSC expression was higher (>100-fold) in MDA-MB-231 compared to MCF-7 cells, and this increment is primarily related to an increased expression of the Nav1.5 isoform (± 1800 times higher in MDA-MB-231 cells). Considering the overall mRNA expression, the expression of Nav1.5 isoform corresponded to about 82% in highly metastatic cells. MDA-MB-231 cells also exhibited high levels of Nav1.7, whereas the expression levels of Nav1.6 channels were relatively low in both cell lines [95]. Pharmacological blockage or upregulation of VGSCs may treat certain types of cancer, especially cases in which Na^+^ channels regulate malignancy [39]. Due to the positive correlation between Na^+^ channel expression and cancer invasion, Na^+^ channel blockers may represent promising new therapies [96,97,98].

## 4. Scorpion Toxins and Their Applications in Cancer Therapy

The study of scorpion venoms was popularized in the 50s, when the venomous components were identified as protein compounds. These proteins, now known to be neurotoxins, were isolated and chemically characterized from many species of scorpions [99]. Currently, knowledge of the structural and functional characteristics of several of these toxins have been used to develop treatments for poisoning victims. This knowledge also enabled other significant advances in biotechnology and pharmacology [100]. Although scorpion poisoning has negative repercussions, scorpion venom is considered by many scientists as a rich source of pharmacologically active compounds. Many studies of toxins have yielded active molecules for the development of treatments for various diseases [99,100,101,102]. 

**Figure 2:**
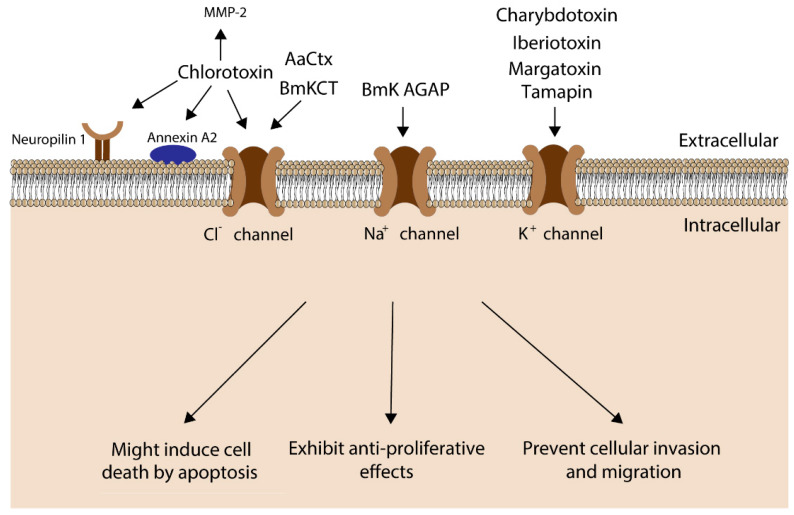
Scorpion neurotoxins and their biological effects in cancer cells.

Relationships between ion channels and cancer progression, in particular, indicate novel clinical applications of scorpion venoms. Indeed, the many peptides and proteins found in scorpion venoms display favorable properties, including high specificity, good permeability, and stability in cancer cells. Several active molecules with anticancer activities like proliferation inhibition, apoptosis promotion and cell migration and invasion reduction, have been isolated from scorpion venoms, identifying scorpion neurotoxins as potential novel cancer therapeutics [103,104,105,106] (Figure 2).

## 5. Anti-Proliferative and Cytotoxic Scorpion Toxins to Cancer Cells 

### 5.1. Chlorotoxin (ClTx)

ClTx [107], isolated from the venom of the giant yellow Israeli scorpion *Leiurus quinquestriatus hebraeus*, is a 36-amino-acid peptide with four disulfide bridges. Structural analysis by solution NMR indicates the presence of an alpha-helix linked by three disulfide bridges to antiparallel β-sheets. The cysteine pattern is C_1_–C_4_, C_2_–C_6_, C_3_–C_7_ and C_5_–C_8_ [107,108]. ClTx inhibits chloride influx in glioma cells, showing little or no activity in normal cells from the same tissue [109]. Another study links ClTx to the matrix metalloproteinase 2 (MMP-2), which is involved in tumor invasion and is overexpressed in glioma. ClTx effectively binds to MMP-2, preventing the gelatinase activity of MMP-2 that changes Cl^−^ currents by internalizing channels [110] (Table 1).

The identification of glioma cells by radiation in Phase I preclinical and clinical studies was obtained using a synthetic version of ClTx coupled to iodine-131 (^131^I-TM-601). It was observed that an intracavitary dose of ^131^I-TM-601 was safe and that the drug interacted with malignant glioma with high affinity for long periods of time [111]. Jacoby and collaborators reported the anti-angiogenic properties of TM-601 co-administered with bevacizumab, revealing that this interaction potentiated the anti-angiogenic effects of bevacizumab more than ten-fold [112].

ClTx has also been used in a liposome delivery system to treat the metastatic breast cancer cell line 4T1 and 4T1 tumors in BALB/c mice. ClTx increased drug delivery to metastatic breast cancers, increasing toxicity and antimetastatic effects [113].

The protein annexin A2, which is present on the surface of some human cancer cell lines and human umbilical vein endothelial cells, was also identified as a receptor for Cltx. Accordingly, binding of TM601 to the surface of Panc-1 pancreatic tumor cells is dependent on the expression of annexin A2 [114]. Recently, using a peptide-drug conjugate (ER-472) composed of ClTx linked to cryptophycin, it was demonstrated that the endocytic receptor on tumor and endothelial cells neuropilin-1 (NRP1) is a novel ClTx target which increases drug uptake leading to an enhanced antitumor activity [115].

The Pt(IV) compound cis, cis, trans-[PtCl_2_(NH_3_)_2_(succinate)_2_] (Pt(IV) complex 1) was coupled to ClTx (Pt-ClTx conjugate) and tested against cervical (HeLa), breast (MCF7), and lung (A549) cancer cells. Results suggested that cervical cancer cells can be treated by this conjugate. It was observed an increment in the cytotoxicity of the Pt-CTX conjugate on HeLa cells. The Pt(IV) complex 1 is 200 times less cytotoxic than cisplatin (a powerful anticancer drug) and it is only 4 times less cytotoxic as a Pt-ClTx conjugate. The cytotoxicity effects of this conjugate indicate that a Pt-ClTx construct might be of interest as a cancer-targeting drug [116].

Another recent application of chlorotoxin is to allow the surgeon to visualize the tumor during surgical resection. A fluorescent nano imaging agent (NIA) with high specificity for U87MG glioma cells was synthesized. The NIA is composed by polymalic acid with chlorotoxin for tumor targeting, indocyanine green for fluorescence and the tri-leucin peptide as a fluorescence enhancer. This method for fluorescence guided resection of glioblastoma was able to significantly improve the precision of tumor removal [117].

### 5.2. AaCtx (Androctonus Australis Chlorotoxin)

The AaCtx is a “chlorotoxin-like” peptide, sharing 70% similarity with ClTx. Isolated from *Androctonus australis* scorpion venom, AaCtx prevents the invasion and migration of human glioma cells. Interestingly, AaCtx has no effects on glioma cell adhesion to extracellular matrix proteins. Notably, the activity of AaCtx was lower than that reported for ClTx. The AaCtx was also tested by its intracerebroventricular injection to mice and no toxic symptoms up to 1 μg doses were observed [118] (Table 1).

The scorpiontoxins AaCTx and ClTx target the matrix metalloproteinase-2 (MMP-2) inhibiting the glioblastoma cell invasion. Othman and collaborators using molecular dynamics, molecular modeling and MM-PB(GB)SA free energy estimation report the interaction mode of ClTx/AaCTx with MMP-2. It was predicted that these scorpiontoxins act on an exosite of MMP-2 comprising mainly residues from the collagen binding domain. This particular interaction could be explored to increase the selectivity toward MMP-2 [119].

### 5.3. BmKCT

The venom of the scorpion *Mesobuthus martensii* Karsch (*Buthus martensii* Karsch) possesses one chlorotoxin-like peptide, called BmKCT, which was identified from a cDNA library made from the venom glands [120]. Similar to ClTx, BmKCT specifically binds glioma cells, inhibiting growth, proliferation, and metastases (*in vivo*, *in vitro*, and *in situ*), with no effect on normal cells [121] (Table 1). Glioma cells selectively express voltage-activated chloride current channels, and potentials activated at >45 mV showed pronounced outward rectification [122]. These cells were sensitive to both ClTx and BmKCT [103].

### 5.4. BmK AGAP

Many different biologically active peptides have already been isolated and characterized from scorpion venoms, and in particular the *Mesobuthus martensii* Karsch (*Buthus martensii* Karsch) venom has been extensively studied because of its use in Chinese traditional medicine for thousands of years [123]. Among the identified peptides, an antitumor–analgesic peptide (BmK AGAP) was obtained from a cDNA library of *B. martensii* scorpion venom [124] (Table 1). BmK AGAP has antitumor activity in mouse S-180 fibrosarcoma and in mouse Ehrlich ascites models, but it also has analgesic activity demonstrated in a mouse twisting model and a hot-plate procedure [124]. Recombinant BmK AGAP (rAGAP) inhibits the proliferation of human glioma cell SHG-44 and rat glioma cell C6, and suppresses the migration of SHG-44 cells during wound healing. rAGAP probably inhibits proliferation by blocking cell cycle progression through G1 to S phase by down-regulating the expression of CDK2, CDK6, and p-RB. The protein levels of anti-apoptotic protein BCL-2, nuclear factor NF-κB/p65, p-AKT, p-p38, p-Erk1/2, p-c-Jun, and VEGF were decreased in cells treated with rAGAP and active cleaved-caspase 3, 8 and 9 were not observed. It was also observed that the mRNA expression of Nav1.5 voltage-gated channel and MMP-9 were down-regulated in dose-dependent manner. These results indicate that rAGAP affects BCL-2, NF-κB/p65, AKT, and MAPK signaling pathways, that might inhibit SHG-44 cells proliferation and migration, but the apoptotic signaling pathways were not activated [125] (Table 1).

The rAGAP peptide increases apoptosis and inhibits the proliferation of human colon adenocarcinoma SW480 cells [126]. This toxin is capable of upregulating the expression of p27 leading to an arrest in G1 phase of the cell cycle. It also suppresses the activation of Bcl-2, phosphatidylinositol 3-kinase (PI3K) and phospho-Akt (p-Akt), and enhances the production of Bax and PTEN (Phosphatase and Tensin Homolog) in SW480 cells [126] (Table 1). Recombinant AGAP suppresses the migration and invasion of HepG2 cells (human hepatoma) via a voltage-gated sodium channel (VGSC) β1 subunit, a cell adhesion molecule (CAM), but has no effect in human liver HL7702 cells without the β1 subunit. When the VGSC β1 subunit is overexpressed in HL7702 normal cells, rAGAP inhibits their migration and invasion [127] (Table 1). The treatment of breast cancer cells MCF-7 and MDA-MB-231 with rAGAP inhibits cancer cell stemness, epithelial-mesenchymal transition (EMT), migration, and invasion. This peptide promotes the down-regulation of pentraxin 3 (PTX3), an inflammatory mediator involved in the activation of complement and the regulation of inflammation, through NF-κB and Wnt/β-catenin signaling pathway. A link between the decreased expression of Nav 1.5 channel and the PTX3 decreased expression in breast cancer after rAGAP treatment was suggested [128] (Table 1).

### 5.5. Charybdotoxin (ChTX)

ChTX, a specific Ca^+2^-activated K^+^ channel blocker that consists of a single polypeptide chain of 4.3 kDa, was isolated from the venom of the scorpion *Leiurus quinquestriatus* [129]. Ion channels are essential for cell motility, and, in turn, the motility of tumor cells is associated with angiogenic and metastatic processes [130]. For example, migration of transformed renal epithelial cells (MDCK-F) and human melanoma cells, are K^+^ channel-dependent. Studies testing ChTX on these cell lines showed migration was slowed in a dose-dependent manner [131,132]. Locomotion of NIH3T3 fibroblasts was also inhibited by ChTX due to K^+^ channel blockade [132] (Table 1).

### 5.6. Iberiotoxin (IbTx)

IbTx is a toxin isolated from the scorpion venom of *Mesobuthus tamulus*. It is a blocker of Ca^+2^-activated K^+^ channels, has 37 amino acid residues and displays 68% sequence homology with Charybdotoxin [133].

IbTx showed a decrease in the growth of glioma cell line D54-MG (World Health Organization grade IV, glioblastoma multiforme) in a dose-dependent manner with a half-maximal inhibitory effect at ∼10 nM and maximal growth inhibition after 4–5 days [134] (Table 1). Recently, it was demonstrated that IbTx selectively decrease anchorage-independent growth and tumorigenicity in breast cancer cells expressing β-catenin (UACC893 and MDA-MB-231). IbTX appeared to specifically attenuate tumorigenicity in breast cancer models by transmembrane depolarization and downregulation of β-catenin and (phospho)Akt and HER-2/neu protein levels [135] (Table 1).

### 5.7. Margatoxin (MgTx)

MgTx, an alpha-KTx scorpion toxin, was purified from *Centruroides margaritatus* scorpion venom. It is a 39 amino-acid-long peptide stabilized by three disulfide bridges with high affinity blocking activity on Kv1.3 channels on mammalian cells [136]. MgTx was considered a selective inhibitor of the Kv1.3 channels but it was demonstrated that MgTx inhibits Kv1.2 and Kv1.3 channels with low picomolar affinities (Kd = 11.7 pM for Kv1.3 and Kd = 6.4 pM for Kv1.2) [137]. Anti-proliferative effects were observed on human lung adenocarcinoma A549 cells using MgTx or short hairpin RNA (shRNA) against Kv1.3, suggesting that Kv1.3 channels are involved in the proliferation of this type of cancer cells. MgTx blockage of Kv1.3 also caused a reduction of tumor volume in a xenograft model using nude mice and the injection of 1 nM MgTx promoted no body weight reduction. These results suggest that Kv1.3 channels may serve as a novel therapeutic target for the treatment of lung adenocarcinoma [138] (Table 1).

### 5.8. Tamapin

Tamapin, a member of the α-KTx5 subfamily, is a venom peptide isolated from the Indian red scorpion *Mesobuthus tamulus*. It has 31 amino acid residues, a molecular mass of 3.5 kDa and is a selective blocker of small conductance Ca^2+^-activated K^+^ channels (KCa2 channels), exhibiting a remarkable selectivity for KCa2.2 (tamapin has IC_50_ = 42 nM, 24 pM, and 1.7 nM for KCa2.1, KCa2.2, and KCa2.3 respectively). Tamapin shares high amino acid sequence similarity with two other KCa2 channel toxins from scorpion venoms, 77% with scyllatoxin and 74% with PO5 [139]. It was observed that recombinant Tamapin induced cell death by apoptosis on Jurkat E6-1 and human mammary breast cancer MDA-MB-231 cells, which express the KCa2.2 channels constitutively, with IC_50_ of 13.6 nM and 0.9 nM respectively. This toxin exhibited low toxicity on human peripheral blood lymphocyte cultures (cell viability: 80% to 88% at the maximum concentration employed). It is suggested that this toxin may serve as an antiproliferative agent in KCa2.2 expressing T cells with no adverse action on common T lymphocytes [140] (Table 1).

## 6. Conclusions

This review highlights the variety of pharmacologically active peptides that can be found in scorpions’ venoms. There is a close relationship between cell death processes and the modulation of membrane ion channels that can be addressed as a tool to access and intervene in the carcinogenic processes. Toxins, especially those from scorpion venoms interacting with ion channels, may be proven highly specific molecules against different types of cancer. Studies on the mechanisms of action of these scorpion toxins in cancer cells are clearly just beginning, but the current accumulated knowledge give us hope to find new, less invasive and less toxic therapies to treat cancer patients.

## Figures and Tables

**Table 1:** Scorpion neurotoxins and their biological effects in cancer cells.

Ion Channel	Toxin	Species	Accession Number	Cell Lineage	Biological Effects	References
	Chlorotoxin	*Leiurus quinquestriatus quinquestriatus*	P45639	Human glioblastoma (D54-MG)	Affects glioma cell invasion	[110]
Cl^−^	AaCtx	*Androctonus australis*	P86436	Human glioma (U87)	Prevents the invasion and migration of human glioma cells	[118]
	BmKCT	*Mesobuthus martensii* Karsch	Q9UAD0	Human glioma (SHG-44)	Inhibits glioma cell migration and invasion *in vitro* and *in vivo*	[121]
Na^+^	BmK AGAP	*Mesobuthus martensii* Karsch	G4V3T9	Ehrlich ascites and S-180 fibrosarcoma *in vivo* Human breast adenocarcinoma (MCF7) Human malignant glioma cells (SHG-44) Human colon adenocarcinoma cells (SW480) Human hepatoma cells (HepG2) Human breast adenocarcinoma (MCF-7 and MDA-MB-231)	Might inhibit SHG-44 cells proliferation and migration through BCL-2, NF-κB/p65, AKT, and MAPK signaling pathways Increases apoptosis and inhibits the proliferation of human colon adenocarcinoma SW480 cells Suppresses the migration and invasion of HepG2 cells via a voltage-gated sodium channel (VGSC) β1 subunit Inhibits cancer cell stemness, epithelial-mesenchymal transition (EMT), migration, and invasion in breast cancer cells	[124,125,126,127,128]
K^+^	Charybdotoxin	*Leiurus quinquestriatus hebreus*	P13487	Human A7 melanoma cells	Induces reduction in melanoma cell migration in a dose-dependent manner	[132]
	Iberiotoxin	*Mesobuthus tamulus*	P24663	Human glioblastoma (D54-MG) Human breast adenocarcinoma (MDA-MB-231) Human ductal mammary gland carcinoma (UACC893)	Induces decrease in the growth of glioblastoma multiforme in a dose-dependent manner Might specifically attenuate tumorigenicity in breast cancer models by transmembrane depolarization and downregulation of β-catenin and (phospho)Akt and HER-2/neu protein levels	[134,135]
	Margatoxin	*Centruroides margaritatus*	P40755	Human lung adenocarcinoma (A549)	Exhibits anti-proliferative effects on human lung adenocarcinoma A549 cells and also causes a reduction of tumor volume in a xenograft model using nude mice	[138]
	Tamapin	*Mesobuthus tamulus*	P59869	Human Leukaemic T cell lymphoblast (Jurkat E6-1) Human breast adenocarcinoma (MDA-MB-231)	Might induce cell death by apoptosis	[140]
